# Hipertensão Induzida pela Obesidade

**DOI:** 10.36660/abc.20230391

**Published:** 2023-07-21

**Authors:** Claudio Leinig Pereira da Cunha

**Affiliations:** 1 Universidade Federal do Paraná Curitiba PR Brasil Universidade Federal do Paraná, Curitiba, PR – Brasil

**Keywords:** Hipertensão Arterial, Obesidade

A hipertensão arterial (HA) e a obesidade são duas das doenças mais comuns e frequentemente podem ser interrelacionadas, provocando significativo dano cardiovascular. O excesso de peso tipicamente aumenta a pressão arterial, e a perda de peso usualmente reduz a pressão arterial.^1^ Além de aumentar o risco da HA, o sobrepeso e a obesidade incrementam o risco cardiovascular por meio de efeitos nos lipídios, fibrilação atrial, insuficiência cardíaca, acidente vascular cerebral, resistência à insulina e outros processos cardiometabólicos, assim como promovem maior mortalidade por todas as causas.^2^ Neste número dos Arquivos Brasileiros de Cardiologia, Santos et al.^3^ apresentam estudo de marcadores laboratoriais e clínicos que associam a HA à obesidade em pacientes com indicação de cirurgia bariátrica.^3^

A prevalência de sobrepeso e obesidade tem aumentado significativamente.^4^ Indivíduos com índice de massa corporal (IMC) ≥ 25 kg/m^2^ , entre 1980 e 2013, passaram de 28,8% para 36,9% da população mundial entre os homens e de 29,8% para 38,0% entre as mulheres.^5^ No Brasil, 52,4% da população encontrava-se com sobrepeso em 2014, sendo que 17,9% eram obesos.^6^

A HA é a doença crônica mais prevalente em todo o mundo, afetando aproximadamente um terço da população adulta. A pressão arterial (PA) é mantida por diversos fatores que incluem principalmente o volume intravascular, o débito cardíaco, resistência vascular periférica e a capacidade elástica das artérias.^4^

O aumento da adiposidade, seja avaliado pelo aumento do peso corporal, o IMC ou a circunferência abdominal, é fortemente associado com maior PA e desenvolvimento de HA. No *Nurses’ Health Study* ,^7^ 82.473 enfermeiras com 30 a 55 anos de idade foram seguidas a cada 2 anos por até 18 anos, e observou-se associação do aumento do IMC com o risco de HA. Nesta pesquisa, o ganho de peso também foi associado com aumento do risco de HA, sendo que as mulheres que ganharam de 5,0-9,9 kg e ≥25,0 kg tiveram risco aumentado de 1,7 a 5,2, respectivamente, para desenvolver HA. A fração de novos casos de HA atribuíveis ao sobrepeso e obesidade foi 40% nesta investigação.

No *Framingham Heart Study* os participantes foram seguidos prospectivamente por até 44 anos, e pôde ser estimado que o excesso de peso corporal, incluindo sobrepeso e obesidade, foi responsável por 26% dos casos de HA nos homens e 28% nas mulheres.^8^

O aumento da PA observado na obesidade é inicialmente associado com uma elevação do débito cardíaco, sendo mantida uma resistência vascular sistêmica (RVS) relativamente normal. Contudo, obesos normotensos têm o mesmo débito cardíaco, mas uma RVS inferior à dos magros normotensos. Então, a diferença hemodinâmica entre obesos hipertensos e normotensos é uma elevação da RVS nos hipertensos, uma diferença similar à observada entre magros hipertensos e normotensos.^9^

As alterações hemodinâmicas na obesidade, associadas com anormalidades nos lipídios e no metabolismo da glicose, parecem estar relacionadas com a distribuição da gordura, e não apenas ao peso corporal. Especificamente, os riscos das anormalidades relacionadas à obesidade são maiores na obesidade abdominal.^10^

Numerosos mecanismos têm sido propostos para associar o sobrepeso e a obesidade com a elevação da PA: 1 – Lesão renal (compressão dos rins pela gordura, ativação do sistema renina-angiotensina-aldosterona, aumento da atividade simpática); 2 – Hiperinsulinemia e resistência à insulina (atividade simpática aumentada, aumento da reabsorção renal do sódio, disfunção endotelial, regulação alterada dos receptores da angiotensina II, redução do sistema peptídeo natriurético); 3 – Apneia obstrutiva do sono; 4 – Via leptina-melanocortina; 5 – Suscetibilidade genética.^11^

Conclui-se que a Hipertensão Arterial associada à Obesidade é mais um motivo para dirigirmos todos os esforços possíveis para combater o descontrole do peso que afeta a população.
