# Supplementary material for: Hipertensão Induzida pela Obesidade
Source: Arq Bras Cardiol. 2023 Jul 21;120(7):e20230391. [Article in Portuguese] doi: 10.36660/abc.20230391 (PMC10421596; doi:10.36660/abc.20230391)
Supplement: Translation

# Translation: Obesity-Induced Hypertension

**Authors:** Claudio Leinig Pereira da Cunha

**Affiliations:** 1 Brasil Universidade Federal do Paraná, Curitiba, PR – Brasil

**Keywords:** Arterial Hypertension, Obesity

Arterial hypertension (AH) and obesity are two of the most common diseases and can often be interrelated, causing significant cardiovascular damage. Excess weight typically raises blood pressure, and weight loss usually lowers blood pressure.^1^ In addition to increasing the risk of hypertension, overweight and obesity increase cardiovascular risk through effects on lipids, atrial fibrillation, heart failure, stroke, insulin resistance, and other cardiometabolic processes, as well as promoting higher mortality from all causes.^2^ In this *Arquivos Brasileiros de Cardiologia* issue, Santos et al.^3^ present a study of laboratory and clinical markers that associate AH with obesity in patients with indication for bariatric surgery.^3^

The prevalence of overweight and obesity has increased significantly.^4^ Individuals with a Body Mass Index (BMI) ≥ 25 kg/m^2^ , between 1980 and 2013, increased from 28.8% to 36.9% of the world population among men and from 29.8% to 38.0% among women.^5^ In Brazil, 52.4% of the population was overweight in 2014, with 17.9% being obese.^6^

AH is the most prevalent chronic disease worldwide, affecting approximately one-third of adults. Several factors, including intravascular volume, cardiac output, peripheral vascular resistance, and the elastic capacity of the arteries,^4^ maintain blood pressure (BP).

The increase in adiposity, whether evaluated by the increased body weight, BMI, or waist circumference, is strongly associated with higher BP and the development of AH. In the Nurses’ Health Study,^7^ 82,473 nurses aged 30 to 55 were followed every 2 years for up to 18 years, and an association between increased BMI and the risk of AH was observed. In this research, weight gain was also associated with an increased risk of AH, with women who gained 5.0-9.9 kg and ≥25.0 kg having an increased risk of 1.7 to 5.2, respectively, to develop AH. The fraction of new cases of AH attributable to overweight and obesity was 40% in this investigation.

At the Framingham Heart Study, participants were prospectively followed for up to 44 years, and it could be estimated that excess body weight, including overweight and obesity, was responsible for 26% of AH cases in men and 28% in women.^8^

The increased BP observed in obesity is initially associated with increased cardiac output, and a relatively normal systemic vascular resistance (SVR) is maintained. However, normotensive obese individuals have the same cardiac output but a lower SVR than normotensive lean individuals. So, the hemodynamic difference between obese hypertensive and normotensive individuals is an increase in SVR in hypertensive individuals, a difference similar to that observed between lean hypertensive and normotensive individuals.^9^

Hemodynamic changes in obesity, associated with abnormalities in lipids and glucose metabolism, appear to be related to fat distribution, not just body weight. Specifically, the risks of obesity-related abnormalities are greater in abdominal obesity.^10^

Numerous mechanisms have been proposed to associate overweight and obesity with increased BP: 1 – Renal injury (kidney compression by fat, activation of the renin-angiotensin-aldosterone system, increased sympathetic activity); 2 – Hyperinsulinemia and insulin resistance (increased sympathetic activity, increased renal sodium reabsorption, endothelial dysfunction, altered regulation of angiotensin II receptors, reduction of the natriuretic peptide system); 3 – Obstructive sleep apnea; 4 – Leptin-melanocortin pathway; 5 – Genetic susceptibility.^11^

It is concluded that the Arterial Hypertension associated with Obesity is yet another reason for us to direct all possible efforts to combat the lack of weight control that affects the population.
